# Infectious complications in patients with ventricular assist device HeartMate II

**DOI:** 10.1186/1749-8090-8-S1-P74

**Published:** 2013-09-11

**Authors:** M Konarik, O Szarszoi, I Netuka, J Maly, J Pirk, M Urban

**Affiliations:** 1 Department of Cardiovascular Surgery, Institute for Clinical and Experimental Medicine, Prague, Czech Republic

## Background

Late infections (more than month post implantation) seem to be significant factor of higher morbidity and mortality in patients with implanted mechanical circulatory support (MCS). The aim of our study was to evaluate occurrence of infectious complications (driveline, pump-pocket) in patients with implanted MCS.

## Methods

Data of patients (n=106), whom was implanted MCS HeartMate II in indication “bridge to transplant” from 11.12.2006 to 3.4.2013 were retrospectively analyzed. Infections were diagnosed on the base of macroscopic finding, positive wound cultivation and laboratory markers of inflammation.

## Results

82 (77.4 %) patients were without infection, 7 (6.7 %) patients developed pump-pocket infection and in 17 (16 %) patients occurred driveline infection. Patients were divided according to driveline technical implantation to three groups: 58 patients with classic/direct technique, 12 patients with „C-shape technique- velour coated“ and 36 patients with „C-shape technique - plastic exit site“. Driveline infection was observed in 13 patients with direct technique (0.28 patient per year), in 3 „velour coated“ patients (0.20 patient per year) and in 1 patient with „plastic exit site“(0.068 patient per year). From this group 15 patients were successfully transplanted, 1 device was explanted, 6 patients died (only 2 patients on infection related complications) and 1 patient is currently on waiting list.

## Conclusion

Late infections are the relevant factor of morbidity and mortality. Despite driveline or pump pocket infections 67 % patients were successfully transplanted or explanted after receiving an appropriate treatment. C-shape method of driveline implantation, especially its plastic site variant, seems to be the method of choice.

